# Real-time nanodiamond thermometry probing in vivo thermogenic responses

**DOI:** 10.1126/sciadv.aba9636

**Published:** 2020-09-11

**Authors:** Masazumi Fujiwara, Simo Sun, Alexander Dohms, Yushi Nishimura, Ken Suto, Yuka Takezawa, Keisuke Oshimi, Li Zhao, Nikola Sadzak, Yumi Umehara, Yoshio Teki, Naoki Komatsu, Oliver Benson, Yutaka Shikano, Eriko Kage-Nakadai

**Affiliations:** 1 Department of Chemistry, Osaka City University, Sumiyoshi-ku, Osaka 558-8585, Japan.; 2 Food and Human Health Sciences, Graduate School of Human Life Science, Osaka City University, Sumiyoshi-ku, Osaka 558-8585, Japan.; 3 Institut für Physik and IRIS Adlershof, Humboldt-Universität zu Berlin, Newtonstraße 15, 12489 Berlin, Germany.; 4 State Key Laboratory of Radiation Medicine and Protection, School for Radiological and Interdisciplinary Sciences (RAD-X) and Collaborative Innovation Center of Radiation Medicine of Jiangsu Higher Education Institutions, Soochow University, Suzhou 215123, P. R. China.; 5 Graduate School of Human and Environmental Studies, Kyoto University, Sakyo-ku, Kyoto 606-8501, Japan.; 6 Quantum Computing Center, Keio University, 3-14-1 Hiyoshi Kohoku, Yokohama 223-8522, Japan.; 7 Institute for Quantum Studies, Chapman University, 1 University Dr., Orange, CA 92866, USA.

## Abstract

Real-time temperature monitoring inside living organisms provides a direct measure of their biological activities. However, it is challenging to reduce the size of biocompatible thermometers down to submicrometers, despite their potential applications for the thermal imaging of subtissue structures with single-cell resolution. Here, using quantum nanothermometers based on optically accessible electron spins in nanodiamonds, we demonstrate in vivo real-time temperature monitoring inside *Caenorhabditis elegans* worms. We developed a microscope system that integrates a quick-docking sample chamber, particle tracking, and an error correction filter for temperature monitoring of mobile nanodiamonds inside live adult worms with a precision of ±0.22°C. With this system, we determined temperature increases based on the worms’ thermogenic responses during the chemical stimuli of mitochondrial uncouplers. Our technique demonstrates the submicrometer localization of temperature information in living animals and direct identification of their pharmacological thermogenesis, which may allow for quantification of their biological activities based on temperature.

## Teaser

Nanoparticle quantum temperature sensors detect subcellular heat generation in a multicellular model organism.

## INTRODUCTION

The temperature inside living organisms is a direct measure of their biological activities. A poikilotherm is a temperature-dependent organism. Even a homeotherm shows internal temperature variations under normal physiological conditions, as can be seen in homeostatic thermoregulation (*1*) and energy metabolism (*2*). Submicrometer-scale temperature information should provide deep understanding on cellular and molecular activities; this information has potential applications for the thermal imaging of brain subtissue structures (*3*), thermal visualization of intratumor heterogeneity (*4*, *5*), and thermogenic mapping of adipocytes (*6*). It is, however, challenging to reduce the size of biocompatible thermometers down to submicrometers. Conventional electric thermometers do not have submicrometer-scale resolution, and near-infrared thermography generally helps determine the surface temperature of biological specimens (*7*). Light-emitting nanothermometers, such as thermoresponsive molecular probes (*8*–*10*) and nanoparticles (*11*, *12*), may resolve these technical limitations. They were first developed for in vitro–cultured cells (*8*–*11*) and, recently, in vivo model animals (*12*). The technical challenges of their in vivo applications are improving their long-term robustness, enabling them to follow the relatively slow response of body temperatures for hours (*2*, *12*) and ensuring their nontoxicity at the high dosages necessary for in vivo measurements.

The nanodiamond (ND) quantum thermometers considered in the present study have emerged as a promising candidate (*13*), exhibiting excellent robustness (*14*–*16*), ultralow toxicity (*15*, *17*), various functionalized surfaces (*18*, *19*), and quantum-enhanced high sensitivity in living cells (*20*, *21*). The sensor reads temperature as a frequency shift of the optically detected magnetic resonance (ODMR) of nitrogen-vacancy (NV) defect centers, which mainly originates from thermal lattice expansion (*22*). The NV sensory core is deeply embedded in the diamond lattice and immune to various biological environmental factors (*23*–*25*). Implementing this quantum sensor in more complex organisms will enable monitoring of their site-specific thermal activities in real time. However, these in vivo applications are technically challenging. Multicellular model organisms such as *Caenorhabditis elegans* worms require a chamber that can hold a millimeter-scale body, and specimens must be rapidly assessed to preserve their physiological condition. ND quantum thermometers move much faster than in cultured cells even if the body is immobilized, necessitating the use of a fast particle-tracking algorithm. Furthermore, the positional movement of NDs and the complex body structure cause substantial fluctuation in the detected fluorescence intensity, which is likely to cause temperature measurement artifacts. The individual technical solutions and their effective system integration are the key to the success of in vivo ND quantum thermometry.

## RESULTS

**Fig. 1:**
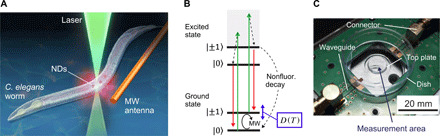
Real-time ND thermometry of *C. elegans* worms. (**A**) ND quantum thermometers probing inside the worms. NDs are incorporated in the worms. ODMR of NV centers can be observed by applying a green laser and microwave excitation. (**B**) Simplified energy diagram of the excited and ground states of NV centers with the associated electron spin states. Green and red arrows indicate the laser excitation and fluorescence, respectively. Microwave (MW) excites the spin state ∣0〉 → ∣± 1〉 in the ground state, which are separated by temperature-dependent zero-field splitting [*D*(*T*)]. The following optical excitation initializes the spin state to the ground state ∣0〉 through nonfluorescent decay (dashed arrow) from the excited state ∣± 1〉. (**C**) Photograph of the antenna-integrated glass-bottom dish docked on a coplanar waveguide on a printed circuit board. Dish contains agar pad and top glass plate. A worm specimen (1 mm in length and 70 μm in width) is placed on an agar pad in the measurement area (see also fig. S1I for schematic configuration). Note that all the enclosure covers and insulating materials were removed for the visual clearance. Photo credit: M. Fujiwara (Department of Chemistry, Osaka City University).

Our in vivo thermometry system is based on a confocal fluorescence microscope equipped with a microwave irradiation setup (Fig. 1A). The ODMR of NV centers can be measured as a decrease in the laser-induced fluorescence intensity when spin-resonant microwave excitation is applied, because the spin excitation activates the nonfluorescent relaxation pathway from the excited state to the ground state (Fig. 1B). Our sample chamber is a disposable antenna-integrated, glass-bottom dish that allows large-area optical access (12-mm diameter) and easy handling (Fig. 1C) suitable for delicate samples such as stem cells (*26*). Quick docking of the dish with a coplanar waveguide on the circuit board shortens the preparation time before the thermometry measurement; a typical preparation time is 15 min from the pick-and-place of the worm to the thermometry experiment. This helps preserve the viability of the worm and facilitates accumulation of large data samples.

**Fig. 2:**
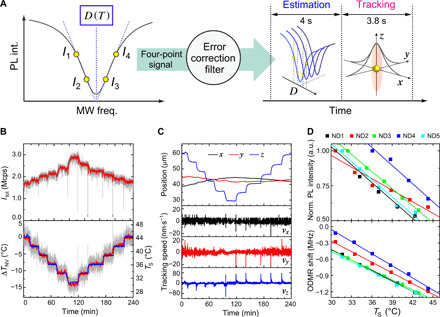
Performance test of error-corrected ND quantum thermometry with fast particle tracking and determination of NDs’ temperature dependence of zero-field splitting and fluorescence intensity under dynamic focal position movement. (**A**) Experimental scheme for the four-point quantum thermometry with error correction filter and particle tracking. *D*(*T*) is estimated from the spin-dependent fluorescence intensities *I*_1_ to *I*_4_ at the four frequency points on the ODMR spectrum. This four-point signal contains an optical power–dependent artifact of NV centers and is subjected to the error correction filter to obtain a correct temperature estimate under the optical fluctuation. The estimation and tracking are sequentially performed to measure the temperature of mobile NDs. (**B**) Time profiles of total photon counts *I*_tot_ (top) and Δ*T*_NV_ (bottom) over the stepwise temperature variation of *T*_S_. Δ*T*_NV_ are calculated with *dD*/*dT* = −65.4 kHz ⋅ °C^−1^, which was experimentally determined in Fig. 2B. Gray indicates Δ*T*_NV_ of every 1 s; red, moving average of 20 sampling points; blue, *T*_S_. (**C**) Corresponding time profiles of ND position (position of the piezo stage) in the *xyz* axes and the tracking speed (first derivative of the positional plot). (**D**) Temperature dependence (*T*_S_) of normalized PL intensity (top) and ODMR shift (bottom) of five NDs on coverslips. a.u., arbitrary units.

Furthermore, our system effectively integrates fast particle tracking and real-time high-precision temperature estimation from the ODMR shift of NV centers. In particle tracking, the system measures the ND fluorescence intensity along the microscope *xyz* axes and focuses on the respective fluorescence maximum every 4 s (a shorter tracking interval is possible), during which temperature is estimated with a sampling time of typically 0.5 to 1.0 s (Fig. 2A). While there are several quantum thermometry methods (*20*, *27*–*30*), we developed a system based on the four-point ODMR measurement protocol (*20*). This estimation implicitly assumes that photon counts registered at all four selected frequencies are linearly scaled to the changes in the detected fluorescence intensity. However, we found that each photon count number exhibits photoresponsivity differences of ∼0.5% (see fig. S2), which actually creates substantial artifacts in the frequency shift estimate (i.e., ∼300 kHz corresponding to several degrees Celsius), particularly in the low-photon regime. This artifact most probably originated from the optical power–dependent asymmetry of the ODMR spectrum and should be corrected for the precise measurement of temperature in optically dynamic environments such as living organisms [see (*31*) for the details of the asymmetry]. We therefore implemented an error correction filter in the four-point method to cancel these effects.

To evaluate the system performance of our real-time quantum thermometry with particle tracking and error correction filter, we measure ND temperatures during step-like thermal events, because sudden changes in temperature cause large defocusing of focal spots and the associated fluctuations in fluorescence intensity; therefore, these tests can demonstrate how fast and precisely our system can track and measure the temperature. Figure 2B shows the time profiles of the total photon counts (*I*_tot_) and the temperature estimate of the NDs (Δ*T*_NV_) when the temperature of the sample (*T*_S_) is varied from 44.3°→30.4°→ 44.3°C in steps of ∼2.8°C (see Materials and Methods for details on how to vary and calibrate *T*_S_). Our system now accurately provides Δ*T*_NV_ corresponding to *T*_S_, while the focal position moves substantially, particularly along the *z* axis over 30 μm (Fig. 2C). The step variation of 3°C causes a *z* positional shift of 6 μm for 3 to 4 min, but the tracking speed is fast enough to follow −105 nm · s^−1^ dynamics at 96 min (Fig. 2C). In addition, Δ*T*_NV_ clearly demonstrates anticorrelation with *I*_tot_ as reported previously (*32*). A statistical study on this type of temperature dependency determines the means and SDs for $I_{\text{tot}}^{-1}\;dI_{\text{tot}}/\mathrm{dT}=-3.9\pm 0.7\%\cdot {}^{\circ}C^{-1}$ and *dD*/*dT* = −65.4 ± 5.5 kHz · °C^−1^ (Fig. 2D), which slightly differ from the previously reported values (see Discussion). The variation of *dD*/*dT* results in a Δ*T*_NV_ error of ∼8% of the measured Δ*T*_NV_ (see Materials and Methods for the error propagation). The temperature precision and accuracy are ±0.29° and <0.6°C, respectively, giving a sensitivity of 1.8°$C/\sqrt{\text{Hz}}$ (see Materials and Methods and fig. S4A).

**Fig. 3:**
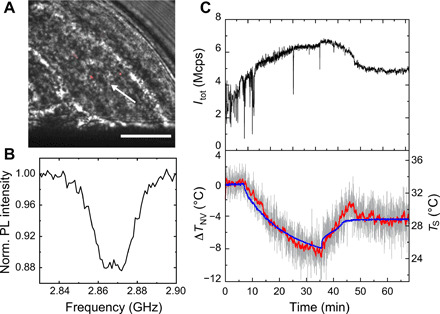
In vivo temperature measurement in *C. elegans* worms with environmental temperature changes. (**A**) A red-gray merged photo of NDs in the worm. Red scale, red fluorescence; gray scale, bright field. The white arrow indicates the ND used for the temperature measurements. The black shadow seen at the bottom part of the image is the microwave linear antenna. Scale bar, 20 μm. (**B**) CW-ODMR spectrum of the ND. (**C**) Time profiles of *I*_tot_ (top) and Δ*T*_NV_ (bottom) during temperature change. Gray indicates Δ*T*_NV_ reading every 1 s; red, moving average of 20 sampling points; blue, *T*_S_. Δ*T*_NV_ is calculated with *dD*/*dT* = −65.4 kHz ⋅ °C^−1^.

Having established robust and accurate thermometry for real-time operation, we test the local temperature monitoring in live worms while applying a temperature shock in the context of a thermosensation study (*33*). Figure 3A shows a picture of the NDs inside worms that are anesthetized and placed near the microwave antennae. These NDs are highly water dispersible by the surface functionalization of polyglycerol (PG) (*34*) and are introduced by microinjection into the gonads (see Materials and Methods) (*17*). Figure 3B is a continuous-wave (CW)–ODMR spectrum of a single ND, which is denoted by the arrow in Fig. 3A. Figure 3C shows the time profiles of *I*_tot_ and Δ*T*_NV_ over a period of 1 hour during a temperature change of *T*_S_. We begin measurements for *T*_obj_ at 33.2°C and decrease it to 25.3°C at 6 min. It is subsequently set to 28.6°C at 35.2 min. Δ*T*_NV_ accurately gives the temperature change between the two stationary states of 33.2° and 28.6°C. The in vivo precision and accuracy values are ±0.22°C (gradually varies to 0.31°C) and <0.6°C, respectively (fig. S7), with a sensitivity of 1.4${}^{\circ}C/\sqrt{\text{Hz}}$. Between these two stationary states, the real temperature dynamics inside worms are reflected because Δ*T*_NV_ always lags behind *T*_S_ and exhibits a slightly underdamped response, owing to the finite heat capacity of the microscope objective and worm surroundings including the agar pads and buffer. *I*_tot_ also shows temperature-induced gradual changes in fluorescence intensities. Particle tracking works effectively during measurement, as can be seen in Fig. 3C; during *t* = 0 to 15 min, the photon counts exhibit frequent spikes originating from ND positional fluctuations of approximately 400 nm for several seconds (fig. S7 and Supplementary Materials). The present demonstration for accurate internal temperature measurement in worms can be directly used to quantify the heat (cold) shock of thermosensory neurons in combination with calcium imaging and optogenetics (*35*, *36*).

**Fig. 4:**
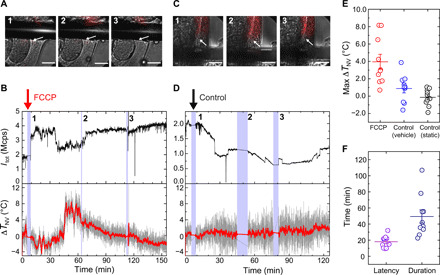
Temperature rise inside *C. elegans* worms by chemical stimulation. (**A** and **C**) Merged photos of NDs during FCCP stimulation (60 μM) and vehicle control experiments. Red scale, red fluorescence; gray scale, bright field. The numbers indicate the timestamps of pictures captured during measurement indicated in (B) and (D). Scale bars, 20 μm. (**B** and **D**) Time profiles of *I*_tot_ (top) and Δ*T*_NV_ (bottom) during FCCP stimulation and vehicle control experiments. The blue shaded regions represent periods when no temperature measurement is performed. The photographs in (A) and (C) were obtained during these periods. Δ*T*_NV_ is calculated with *dD*/*dT* = −65.4 kHz ⋅ °C^−1^ for both types of experiments. (**E**) Statistical plots of the maximum Δ*T*_NV_ for FCCP stimulation (red), vehicle control (blue), and static control (black, no solution added). *n* = 10 for all data. The mean values with SE are 4.0 ± 0.9°C, 0.9 ± 0.5°C, and −0.1 ± 0.3°C for the FCCP, vehicle control, and static control experiments, respectively. All measurements were performed at a constant temperature of 23°C with fluctuation less than 0.25°C (see fig. S4B for the stability). The probed NDs were located within 20 μm of the antenna with a mean of 8.9 μm. (**F**) Latency and duration of the temperature-increase responses of Δ*T*_NV_ for the FCCP stimulus, whose means with SEs are 18.1 ± 2.3 and 49.4 ± 8.22 min, respectively.

As we have demonstrated the capability of quantifying the local temperature inside live worms, we use the ND thermometry for in vivo thermogenic studies. In particular, we monitor the internal temperatures of worms under pharmacological treatment to induce nonshivering thermogenesis by using a mitochondrial uncoupler, i.e., carbonyl cyanide *p*-trifluoromethoxyphenylhydrazone (FCCP) (*8*, *37*). Figure 4 (A and B, respectively) shows a sequence of microscopic images of NDs in worms when the worms are stimulated by FCCP, and the time profile of Δ*T*_NV_ of the arrow-indicated ND. In this temperature response curve, at *t* ∼ 7 min, the FCCP solution was added to the culture medium. At *t* ∼ 32 min, Δ*T*_NV_ starts to gradually increase; at *t* ∼ 48 min, an additional increase is observed as the total change reaches 4° to 7°C. This temperature rise lasts about 80 min until *t* ∼ 120 min. The observed anticorrelation between *I*_tot_ and Δ*T*_NV_ also supports the temperature increase (*t* = 40 to 70 min). During the stimulation, the NDs slowly move several micrometers over an hour, which corroborates the results of separate experiments in which the NDs were continuously observed under a commercial microscope (movie S1). As a control experiment for the stimulation, we test a vehicle solution (Fig. 4, C and D). At *t* ∼ 6 min, the vehicle solution is added; however, Δ*T*_NV_ exhibits a flat response and does not exhibit any noticeable increase.

To further confirm the temperature increase by FCCP, we measure the number of ND-labeled worms for the FCCP treatment and control experiments, as shown in Fig. 4E (see Materials and Methods and fig. S9 for details on quantifying the response curves). We observe the clear tendency for internal temperature increase when stimulated by FCCP, compared with the vehicle control experiments in which only the vehicle solution is added. Another control experiment in which no buffer solution is added and Δ*T*_NV_ is only monitored statically (static control; see fig. S4D for the representative profile) indicates that droplet addition causes the fluctuation of Δ*T*_NV_ at a certain level via either temperature change or ODMR shift artifacts; however, it is insufficient to create the observed ODMR shift with FCCP addition, further confirming the temperature increase by FCCP (see also fig. S10 for the three distinct types of clusters in the correlation plots of maximum Δ*T*_NV_ and integrated response curve areas). As characteristic parameters for identifying the response curves, we summarize the latency and duration of the observed temperature responses in Fig. 4F. The wider distribution of duration when compared with that of latency may be caused by insufficient controllability of the FCCP concentration or dehydration-induced viability. The quantitative interpretation of these response curves should consider several factors, including data extraction from the temperature time trace, particle inhomogeneity of *dD*/*dT* of NDs, and lack of information on thermogenesis in *C. elegans*, which are discussed in Discussion.

## DISCUSSION

This study demonstrates the effectiveness of integrating four-point ODMR measurement with a quick-docking sample chamber, fast particle tracking, and an error correction filter. Our integrated system enables technically demanding experiments necessary for real-time quantum nanothermometry in complex multicellular organisms. Direct extensions of the present results are the recent ND-labeled animal models, including zebrafish and fruit fly models (*15*). Monitoring temperature during their embryogenesis and metamorphosis can provide information about ultradian rhythms or thermoregulation of the developmental processes (*38*, *39*). In embryos of *C. elegans*, the microscopic mechanism of the cell division cycle has been recently addressed by measuring the local temperature using ND quantum thermometry (*40*). Temperature monitoring of migrating cells is also anticipated, as the tracking speed of our system exceeds 100 nm · s^−1^, which covers the crawling speeds of various cells (*41*). For example, by combining the proposed system with intravital microscopy (*42*), it would be possible to probe site-specific temperatures of tumors, organs, and model organoids when ND-labeled cells migrate into them; such a process may be used to analyze the dynamics of cancer metastasis or stem cell engraftment.

To implement the ND quantum thermometry more deeply into such biological analyses, we note that some technical challenges remain: (i) methods for correct determination of *dD*/*dT* are needed, (ii) measurement artifacts must be more thoroughly understood, and (iii) methods for combining thermometry with other biological assays need to be established. First, we determined *dD*/*dT* = −65.4 ± 5.5 kHz · °C^−1^ by measuring the step-like temperature profiles for the five NDs (Fig. 2). This value is relatively smaller than the previously reported values of approximately −74 kHz · °C^−1^ (see Supplementary Materials for the comparison of *dI*_tot_/*dT* with previous reports) (*43*–*45*). Although the exact reason for this discrepancy is not known, it may be caused by material inhomogeneity and calibration difficulty. A value of −74 kHz · °C^−1^ was previously determined for the NV centers in bulk diamonds, with a sample variation from −71 to −84 kHz · °C^−1^ (*43*–*45*). It is known that *dD*/*dT* has greater inhomogeneity in NDs because of crystal strains and surface states (*46*, *47*), and it may be further affected in different types of ND samples. The calibration difficulty may also be a cause of the discrepancy. We calibrated the sample temperature (*T*_S_) by using a tiny flat thermistor tightly attached to the coverslip with heat-conducting tape. While this is the basic method for measuring the surface temperature, *T*_S_ does not necessarily exhibit the exact temperature that NDs are feeling on the coverslip surface. The ODMR measurement method adds complexity when generalizing results with other reported data because it also affects the accuracy of temperature (namely, the *dD*/*dT* value). For example, the three-point ODMR method (*27*, *30*) provides temperature estimates that depend considerably on the spectral shape of ODMR (*31*). It correctly provides the ODMR shift for a perfect single Lorentzian shape, but, in most cases, its estimates are too large (by a factor of 2.2 compared with the four-point method) depending on the actual spectral shape and selected frequency points. It is necessary to take account of the measurement methods when quantitatively determining and comparing *dD*/*dT*.

Second, a thorough understanding of measurement artifacts is still necessary. Although we clearly observed a temperature increase inside worms via the time profiles of Δ*T*_NV_ (Fig. 4) and their correlation plots with the integrated response curve areas (fig. S10), its quantitative interpretation is challenging at present because of incomplete understanding of the sensory behaviors of NV centers in live worms and the lack of complete information regarding *C. elegans* thermogenesis. The observed drifts and jumps in Δ*T*_NV_ (also confirmed as shifts in CW-ODMR spectra, as in fig. S8) cannot be explained well at present because the spin properties of NDs in living worms have not been well studied. The mean values of Δ*T*_NV_ from the FCCP stimulation and control experiments cannot be directly compared, for example, by subtracting the two cases, because the time profiles of Δ*T*_NV_ are very different. For example, the present analysis shows +0.7°C at 23 min for the vehicle control in Fig. 4D, although its interpretation is not straightforward. The possible variation of *dD*/*dT* can further affect the temperature values. While this study uses *dD*/*dT* = −65.4 kHz · °C^−1^, as mentioned above, a *dD*/*dT* range of −50 to −100 kHz · °C^−1^ has been reported (*46*). Without determining *dD*/*dT* for each measured ND in situ (*48*), a quantitative interpretation of the temperature response curve would be challenging.

Third, the combination of ND thermometry with other biological assays will be required for relating the observed local temperature data to physiological understanding. For instance, it is still unclear how physiological thermogenesis, including shivering and nonshivering thermogenesis, occurs in live worms. The slight and relatively short-lived temperature increase in the control experiments may be ascribed to shivering thermogenesis if not caused by measurement artifacts. In this case, one naturally needs to measure multiple parameters, including oxygen consumption rate (*49*) and mitochondrial membrane potential (*50*), to comprehensively understand the observed phenomena to confirm the shivering thermogenesis.

Last, it should be noted that detecting endogenous heat generation at the subcellular level has prompted extensive debate over the years (*51*, *52*), as temperatures inside cultured cells measured by fluorescent probes show a large inconsistency with expected temperature increases (10^−5^ °C) calculated based on the cellular heat generation rate (10 to 100 pW per cell) and heat transfer. However, subcellular temperature detection of endogenously generated heat has been increasingly reported using other techniques such as label-free Raman microscopy (*53*) and organic fluorescent probes in combination with oxygen consumption rate assays (*54*). For *C. elegans* worms, a micromachined calorimeter recently measured the endogenous heat generation for a few hundred worms when stimulated by relatively low-concentration FCCP of 30 μM, which gave a heat generation rate of ∼ 5 nW per worm (*37*). While there are differing experimental parameters and unknown factors among the studies, including FCCP concentration, treatment time, population inhomogeneity of temperature responses, and uncertainty regarding the heat conductivity of worms, the discrepancy between the observed temperature rise and the estimation decreased by a factor of 10^1^ to 10^2^ compared with the discrepancies observed with cultured single cells.

In conclusion, we have developed an ND quantum thermometry system that can measure the temperatures of mobile NDs inside live adult worms of *C. elegans* with a precision of ±0.22°C. This in vivo thermometry system is equipped with a quick-docking sample chamber, fast particle tracking, and an NV error correction filter to accurately measure the temperature dynamics inside the worms during environmental temperature changes. By using this system, we have determined the temperature increase caused by the worm’s thermogenesis under the treatment of mitochondrial uncoupling stimuli. The results highlight the potential to probe subcellular temperature variations inside living organisms and may allow for the study of the submicrometer thermal effects to biological processes.

## MATERIALS AND METHODS

### Real-time ODMR microscopy setup and temperature calibration

The thermometry system is based on a confocal fluorescence microscope equipped with microwave control for ODMR measurements (fig. S1A). A CW 532-nm laser was used for the excitation of NDs with an intensity in the range of 1 to 10 kW·cm^−2^ to adjust *I*_tot_ to be around 2 mega-counts per second (Mcps). An oil immersion microscope objective with a numerical aperture of 1.4 was used for both the excitation and the fluorescence collection. The NV fluorescence was filtered by a dichroic beam splitter (Semrock, FF560-FDi01) and long-pass filter (Semrock, BLP01-635R-25) to remove the residual green laser scattering and in vivo background fluorescence. The fluorescence was then coupled to an optical fiber (Thorlabs, 1550HP) to be detected by an avalanche photodiode (Excelitas, SPCM-AQRH-14). Samples were placed in the incubation chamber, and the chamber was mounted on a piezo stage that enabled raster scanning and particle tracking. The output from the avalanche photodiode was fed to a data acquisition board system (National Instruments, USB-6343 BNC and USB-6229 BNC), where four of the six equipped counters were used.

To implement both the CW- and four-point ODMR measurements, a stand-alone microwave source (Rohde & Schwarz, SMB100A) and three USB-powered microwave sources (Texio, USG-LF44) were connected to an SP6T switch with a switching time of 250 ns (General Microwave, F9160). The microwave was then amplified (Mini-Circuits, ZHL-16W-43+) and fed to a microwave linear antenna (25-μm-thin copper wire) in our disposable antenna-integrated glass-bottom dish. The penetration depth of the microwave magnetic field into water media (or agarose pads, worm body) was approximately 40 μm (exponential decay length shown in fig. S1D) as calculated by the finite-element method (COMSOL). We used ε = 1.00 for air and ε = 76.6 − 10.6*i* at 2.8 GHz for water (*55*). The typical microwave excitation power was estimated to be 10 to 50 mW (10 to 17 dBm) at the linear antenna by considering the source output, amplifier gain, and the experimentally determined *S*_21_ of the docking chamber, which provides a microwave magnetic field of more than 2 to 5 gauss within 20 μm of the antenna. This theoretical estimation corresponds to the experimental observation that NDs exhibiting a CW-ODMR depth lower than 0.92 were typically found within 20 μm of the antenna. In the CW-ODMR measurements, APD detection was gated for microwave irradiation on and off using the SP6T switch and a bit pattern generator (SpinCore, PBESR-PRO-300), where the gate width was 200 μs for both gates, followed by a laser shutoff time of 100 μs, resulting in $I_{\text{PL}}^{\text{ON}}$ and $I_{\text{PL}}^{\text{OFF}}$ with a repetition rate of 2 kHz (fig. S1E). Note that an external magnetic field was not applied in this study. In the four-point ODMR measurements, APD detection was gated for the corresponding microwave frequencies (ω_1_ to ω_4_), where the gate width for all four gates was 100 μs, each followed by an interval of 5 μs (fig. S1F). This gated photon counting of the four counters was performed approximately 2380 times for a second. The obtained fluorescence intensity signals at four frequencies were error corrected and subsequently used for the temperature estimation, as described below.

The temperature dependence data of Figs. 2 and 3C were measured for the spin-coated NDs (Adámas Nanotechnologies, NDNV100nmHi10ml, 500 NV per particle) on coverslips (fig. S1H) and the ND-labeled worms in the antenna-integrated glass-bottom dish (fig. S1I). The *T*_S_ was varied via direct heat conduction from the oil immersion microscope objective whose temperature (*T*_obj_) was controlled by a proportional-integral-derivative feedback controller of the foil heater wrapping the objective (Thorlabs, HT10K and TC200; temperature precision, ±0.1°C). The immersion oil was Olympus Type F. *T*_S_ was calibrated in the following manner: (i) a tiny flat Pt100 thermistor (Netsushin, NFR-CF2-0505-30-100S-1-2000PFA-A-4, 5 × 5 × 0.2 mm^3^) was tightly attached to the sample coverslip by a thin layer of silicone vacuum grease between the probe and the coverslip. (ii) The thermistor was completely covered by aluminum tape whose edges were glued to the base coverslip. (iii) In this thermal configuration, *T*_obj_ was varied while monitoring *T*_S_. We obtained the following relation: *T*_S_ = 1.847 + 0.923*T*_obj_ (fig. S3). The thermistor was read by a high-precision handheld thermometer (WIKA, CTH7000; precision, 0.01°C). During the calibration measurement, the room temperature (*T*_air_) was monitored using a data logger (T&D, TR-72wb, precision: 0.5°C), and we confirmed that *T*_air_ fluctuates within only ±0.6°C over 12 hours. Note that *T*_obj_ was monitored directly on top of the foil heater. The temperature stability in the incubator was ±0.25°C over 250 min when measured by the thermistor (fig. S4B) or ±0.4°C over 140 min when measured by NDs on the coverslip in the manner of the four-point method (fig. S4C). The stability was ±0.6°C over 140 min when measured by NDs in worms in the manner of the four-point method (fig. S4D) when the in vivo baseline drift did not occur (fig. S8).

### Four-point analysis of ODMR signals and error correction filter

In the four-point ODMR measurements (*20*), fluorescence intensities at four frequency points (*I*_1_ to *I*_4_ for ω_1_ to ω_4_) on CW-ODMR spectra were measured. To determine these frequencies, we measured the entire CW-ODMR spectral shape and then applied a fit to the sum of two Lorentzian functions to indicate the ODMR spectrum and zero-field splitting *D* (see fig. S1G). Accordingly, the two linear slopes of the ODMR spectrum were recognized via linear fits, and the four frequency points, two on each slope, were uniformly distributed, i.e., equally distanced with δω over the extent of the slopes. Here, ω_−_ and ω_+_ were centered on *D* such that *I*(ω_−_) = *I*(ω_+_). *I*_1_ to *I*_4_ were then given by

$$\begin{array}{c} I_{1}=I(\omega_{-})+\gamma_{1}[-\delta \omega +\delta \beta +\delta T(\frac{\mathrm{dD}}{\mathrm{dT}})],\\ I_{2}=I(\omega_{-})+\gamma_{1}[+\delta \omega +\delta \beta +\delta T(\frac{\mathrm{dD}}{\mathrm{dT}})],\\ I_{3}=I(\omega_{+})+\gamma_{2}[-\delta \omega -\delta \beta +\delta T(\frac{\mathrm{dD}}{\mathrm{dT}})],\\ I_{4}=I(\omega_{+})+\gamma_{2}[+\delta \omega -\delta \beta +\delta T(\frac{\mathrm{dD}}{\mathrm{dT}})] \end{array}$$ (1)

where γ_1_ and γ_2_ depict the slopes of the two linear domains. δβ is an unknown static magnetic field (*20*) but assumed to be zero in the present study. Note that the splitting of the ODMR dip is due to the interference of the ^3^A spin states and lattice strains (*21*, *56*, *57*). The splitting does not affect thermometry precision or accuracy in these four-point measurements. By assuming that ∣γ_1_∣ and ∣γ_2_∣ are equal (see the Supplementary Materials for the error of this simplification), the temperature estimate Δ*T*_NV_ was given by

$$\Delta T_{\text{NV}}=\delta \omega {\left(\frac{\mathrm{dD}}{\mathrm{dT}}\right)}^{-1}\frac{\left(I_{1}+I_{2})-(I_{3}+I_{4}\right)}{\left(I_{1}-I_{2})-(I_{3}-I_{4}\right)}$$ (2)

To correct the systematic errors originating from the photoresponsivity differences between the four counters, the dependence of the counter values (*I*_1_ to *I*_4_) on the NV fluorescence intensity was first measured by controlling the laser power (fig. S2A). Two sets showing the differences between two counters with the same ODMR depth, *I*_1_ − *I*_4_ and *I*_2_ − *I*_3_, were plotted as functions of *I*_4_ and *I*_3_, respectively, and were fitted with second-order polynomials (fig. S2B) as described by

$$\begin{array}{c} I_{3}^{\prime }=I_{3}+[a_{0}+a_{1}I_{3}+a_{2}I_{3}^{2}],\\ I_{4}^{\prime }=I_{4}+[b_{0}+b_{1}I_{4}+b_{2}I_{4}^{2}] \end{array}$$ (3)

Here, $I_{i}^{\prime }$ and *a_k_*, *b_k_* denote the error-corrected photon counts and coefficients of the second-order polynomials for *I*_3_, *I*_4_, respectively; we used $I_{i}^{\prime }$ instead of *I_i_* in Eq. 2. With this error correction, the effect of the systematic error was successfully canceled, as shown in fig. S2C, and a constant real temperature was obtained. Without the error correction, intentional step variations in the photon counts (*I*_tot_) caused artifacts in the estimation.

The total photon count *I*_tot_ was obtained using the following equation

$$I_{\text{tot}}=1.05\times (I_{1}+I_{2}+I_{3}+I_{4})$$ (4)

where the factor of 1.05 was the correction factor accounting for the time interval of 5 μs, during which no photons were detected.

### Particle tracking

For particle tracking, the piezo stage was scanned in the *xyz* directions while measuring the ND fluorescence intensity. The obtained cross sections of the point spread function along the *xyz* axes were fitted with a Gaussian function to determine the *xyz* positions for repositioning. The piezo stage was moved smoothly to the repositioning point by five steps of ∼20 nm every 2 ms. Repositioning took 3 s and was performed every 4 s during the four-point measurement. Note that temperature estimation was performed every 0.5 to 1.0 s during the 4 s. Particle tracking precision was discussed in the Supplementary Materials. Occasionally, ND particles moved beyond the maximal range of particle tracking, particularly in the in vivo experiments shown in Fig. 4 because of the worms’ movements. In these cases, the same ND particles were searched and manually moved back to the focus area based on the wide-field fluorescence image recorded before the movement.

### Determination of precision and accuracy

The accuracy of Δ*T*_NV_ was determined by first adding an offset to Δ*T*_NV_ to match *T*_S_ and by taking the root mean square (RMS) of *T*_S_ − *T*_NV_ (figs. S4A and S7B). The upper bound of the RMS in the steady state was considered to be the accuracy in this study. Note that the fluctuation of *T*_air_ deviated Δ*T*_NV_ from *T*_S_, which overestimated the accuracy value. The precision (σ_p_) was determined by taking the SEs of 20 sampling points of Δ*T*_NV_ that were recorded for 38 s (figs. S4B and S7A). Because this duration comprised 19.4-s integration time (δ*t*_int_) and 18.6-s repositioning time, the sensitivity (η_T_) could be calculated as $\eta_{T}=\sigma_{p}\times \sqrt{2\delta t_{\text{int}}}$. Note that Δ*T*_NV_ in the in vivo experiments shown in Figs. 3 and 4 has a variation of ∼ ±8% propagated from the error of *dD*/*dT*, which can be calculated as

$$\delta \Delta T_{\text{NV}}=\delta D^{\prime }{\left(\frac{\mathrm{dD}}{\mathrm{dT}}\right)}^{-1}\Delta T_{\text{NV}}$$ (5)

where δ*D*′ is the error of *dD*/*dT*.

### Highly water-dispersible PG grafted NDs

ND was grafted with PG according to the method reported previously (*34*) (fig. S5A). In brief, 5 mg of hydroxylated NDs with a median diameter of 168 nm (NDNV100nmOH, 900 NV per particle, Adámas Nanotechnologies) was mixed with 5 ml of glycidol (Aladdin Chemical, Shanghai) and then bath sonicated at 20°C for 30 min. The suspension was magnetically stirred at 140°C for 20 hours. The resulting yellowish gel was cooled to room temperature and diluted with 20 ml of water through bath sonication. After removing unbound PG by centrifugal filtration (Amicon Ultra-15, MWCO 100K) and washing with water several times, the resulting PG-ND (6.8 mg) was recovered and redispersed in water (1.88 g ml^−1^). The PG-ND dispersion showed a particle size of 181 nm in the dynamic light scattering (fig. S5B).

### ND-labeling *C. elegans* worms

The wild-type *C. elegans* strain Bristol N2 was obtained from the Caenorhabditis Genetics Center (Minneapolis, MN). *C. elegans* was maintained using standard techniques (*58*). Young adult hermaphrodites were injected by using the standard procedures (*59*), with some modifications. In brief, glass capillaries (Narishige, ND-1) were pulled using a pipette puller (Olympus, PC-100). Needles were filled with the PG-ND dispersion. They were mounted on a manipulator (Narishige, MN-4) and pressurized through an injection system (Narishige, IM-31). Worms were immobilized on agarose injection pads that were covered with paraffin oil (Wako, Japan). The microscope used for the injection was equipped with differential interference contrast (Olympus, IX73). The PG-ND dispersion was injected into the distal arm of the gonad (fig. S6). The injected worms recovered on bacteria-seeded nematode growth medium plates for more than a day.

### Transfer of worms and adding chemical stimuli

The ND-labeled worms were transferred from the culture dishes onto the agar pads prepared on small pieces of glass substrates (Fig. 1C and fig. S1I). A small aliquot of sodium azide solutions (50 mM) was dropped for anesthesia. The agar pads that held the worms were placed on the antenna-integrated glass-bottom dishes, while the worm position was adjusted near the microwave antenna. FCCP (10 mM; Sigma-Aldrich, Japan) in dimethyl sulfoxide (DMSO) (Wako, Japan) was diluted to 0.6% (v/v) with M9 buffer (5 mM potassium phosphate, 1 mM CaCl_2_, and 1 mM MgSO_4_) to give a 60 μM FCCP solution. The 0.6% (v/v) DMSO/M9 buffer solution without FCCP was used for the control experiment. Small droplets of FCCP solution and vehicle control buffer solution were placed near the agar pads to spontaneously spread into the sandwiched region. These measurements were performed at 23°C with a periodic fluctuation of ±0.5°C every 40 min. For the particle tracking measurement shown in movie S1, a commercial confocal microscope (Leica, Germany) was used with a setup of laser excitation at 552 nm and emission filter of 645 to 700 nm for transmission window.

### Statistical analysis of time profile of Δ*T*_NV_

To quantify the response curves of the increase in temperature, we chose the peak height, integration area, latency, and duration of the response curves as the characteristic parameters. Because Δ*T*_NV_ sometimes exhibits long-term baseline drifts during the measurements and sudden jumps at the time of the droplet addition (either FCCP or control), these artifacts were linearly subtracted as shown in the top panel of fig. S9. These compensated data were low-pass filtered by a Lowess filter with a span of 0.1 to extract the envelope of the response curve (middle panel of fig. S9). The integration area was obtained by integrating the Lowess-smoothed curves. Time points representing 5% increases in integration in the smoothed curve were used to determine latency, and the elapsed time between the 5 and 95% time points was used as the duration. Note that the baseline drifts and jumps of Δ*T*_NV_ only occur in worms and not for NDs on coverslips in air. To obtain further details on these phenomena, we measured the CW-ODMR and fluorescence spectra of NDs before and after the long-term drifts and sudden jumps as shown in fig. S8. The CW-ODMR spectra were shifted in agreement with the four-point measurement data. However, slight changes in spectral shapes were also observed.

## Supplementary Material

aba9636_SM.pdf

aba9636_Movie_S1.mp4

## SUPPLEMENTARY MATERIALS

Supplementary material for this article is available at http://advances.sciencemag.org/cgi/content/full/6/37/eaba9636/DC1

## Appendix

View/request a protocol for this paper from *Bio-protocol*.
