# Supplementary material for: Real-time nanodiamond thermometry probing in vivo thermogenic responses
Source: Sci Adv. 2020 Sep 11;6(37):eaba9636. doi: 10.1126/sciadv.aba9636 (PMC7486095; doi:10.1126/sciadv.aba9636)
Supplement: aba9636_SM.pdf [file aba9636_SM.pdf]

Science Advances
AAAS

advances.sciencemag.org/cgi/content/full/6/37/eaba9636/DC1

Supplementary Materials for

Real-time nanodiamond thermometry probing in vivo thermogenic responses

Masazumi Fujiwara*, Simo Sun, Alexander Dohms, Yushi Nishimura, Ken Suto, Yuka Takezawa, Keisuke Oshimi, Li Zhao, Nikola Sadzak, Yumi Umehara, Yoshio Teki, Naoki Komatsu, Oliver Benson, Yutaka Shikano*, Eriko Kage-Nakadai*

*Corresponding author. Email: masazumi@osaka-cu.ac.jp (M.F.); yutaka.shikano@keio.jp (Y.S.); nakadai@life.osaka-cu.ac.jp (E.K.-N.)

Published 11 September 2020, *Sci. Adv.* **6**, eaba9636 (2020)
DOI: 10.1126/sciadv.aba9636

The PDF file includes:

Supplementary Text
Figs. S1 to S10

Other Supplementary Material for this manuscript includes the following:

(available at advances.sciencemag.org/cgi/content/full/6/37/eaba9636/DC1)

Movie S1

Supplementary materials

Effect of assuming that the two linear slopes of the ODMR spectrum are equal

In the Methods section, to obtain Eq. 1 from Eq. 2, $|\gamma_1|$ and $|\gamma_2|$ were assumed to be equal. However, they are slightly different with a variation of $\sim 5.0\%$. This difference may affect temperature estimates, particularly in the *in-vivo* experiments, because slight changes of the ODMR spectra were observed in worms (Fig. S8).

Temperature dependency of the fluorescence intensity of NV centers in NDs

In the main text, we reported the temperature dependence of the fluorescence intensity of $I_{\rm tot}^{-1}\ dI_{\rm tot}/dT = -3.6 \pm 0.2\ \% \cdot {}^{\circ}{\rm C}^{-1}$. This value is larger than the previously reported values for NDs; the previous study reports the same variation in the fluorescence intensity for the change of $\sim$ 150°C with a large variation of 70–270 °C (*32*). Besides the temperature dependency of the NV centers, the overall optical throughput of the microscopy system may be related to the observed fluorescence variation. The imaging property of the microscope objective is optimized for room temperature at approximately 20 °C. The temperature variation may change the diffraction pattern and the back focal pattern of the laser and fluorescence, which finally affects the optical throughput at the pinhole because of spatial filtering. It should be noted that the fluorescence intensity is strongly affected by the optical transmission inside the worm's body and cannot be used independently as a temperature indicator. The clear observation of the

thermal events in $\Delta T_{\rm NV}$ in live worms is by itself a strong indication of the temperature change in the worms.

Particle tracking precision

Particle tracking precision in this study was mainly limited by the step size of the re-positioning process. For re-positioning, the piezo stage was scanned in the xyz directions with a step size of 32 nm to obtain the point spread function. The one-dimensional cross-section was fitted with a Gaussian function to determine the central peak position. In general, well-isolated single peaks allow for accurate positioning of $\sim$ 20 nm, whereas distorted or double peak functions deteriorate positioning accuracy, particularly in live worms. Furthermore, in live worms, other NDs moving around sometimes get closer to the focus and generate a second peak in the point spread function of the locked ND, which in many cases prohibits particle tracking. Note that particle tracking is a feedback process to maximize the fluorescence counts of NDs that are likely to move away from the focus and can be coupled with variations of fluorescence intensity derived from temperature changes in NV centers depending on feedback protocols. While the present feedback protocol is not significantly coupled with the temperature estimation because of the constant repositioning time, it is somewhat coupled with the thermometry, which becomes significant in the low photon regime of $I_{\rm tot} < 0.5$ Mcps. We therefore adjusted the photon counts to always be larger than this threshold (ideally $I_{\rm tot} > 0.75$ Mcps) to exclude any measurement artifacts.

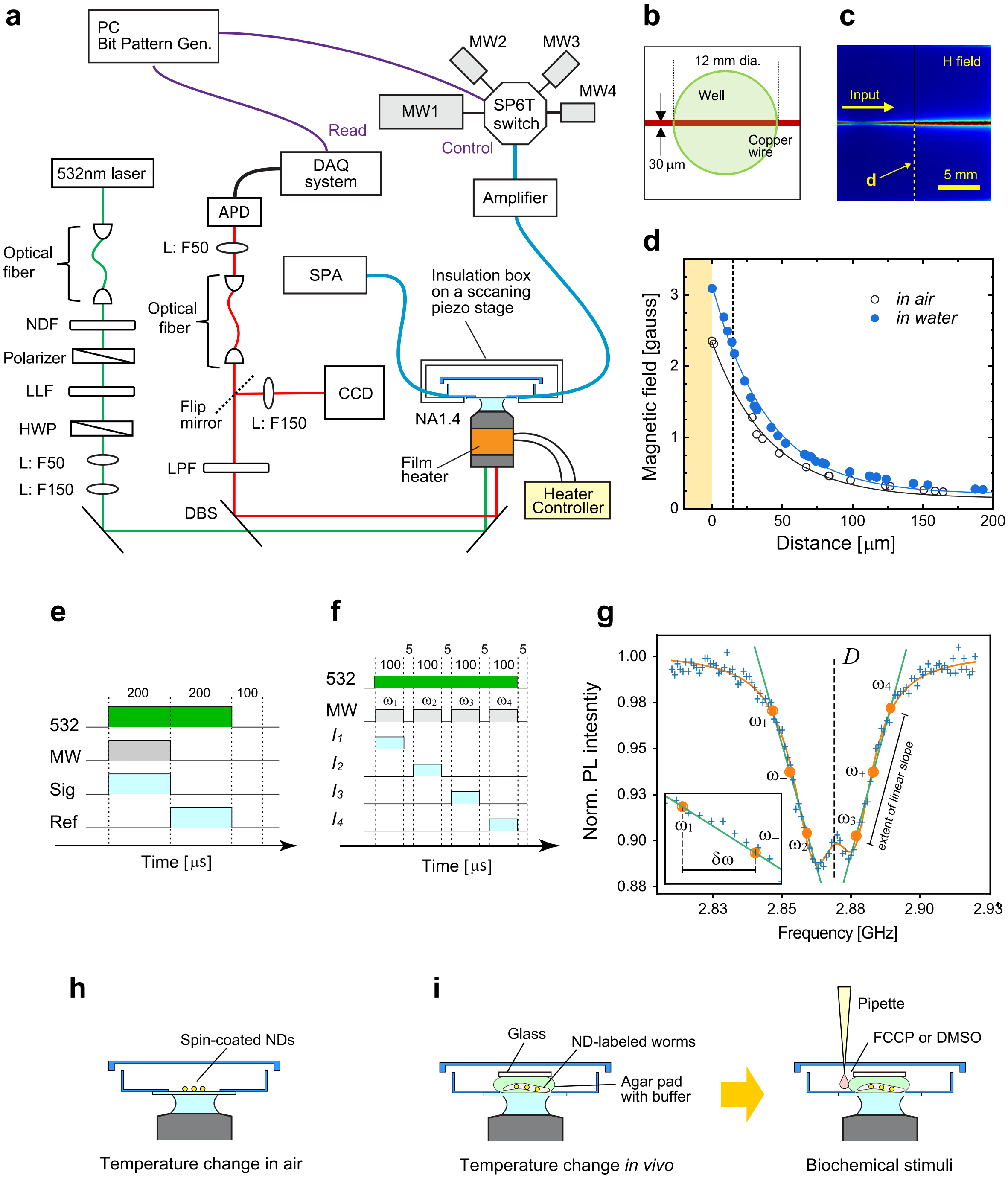
a
PC
Bit Pattern Gen.
MW2
MW3
MW4
MW1
SP6T
switch
Read
Control
DAQ
system
532nm laser
APD
Amplifier
L: F50
SPA
Insulation box
on a sccaning
piezo stage
Optical
fiber
NDF
Polarizer
LLF
HWP
L: F50
L: F150
Flip
mirror
L: F150
CCD
NA1.4
Film
heater
Heater
Controller
LPF
DBS
b
12 mm dia.
Well
Copper
wire
30 μm
c
H field
Input
d
5 mm
d
Magnetic field [gauss]
in air
in water
Distance [μm]
e
200
200
100
532
MW
Sig
Ref
Time [μs]
f
5
100
532
MW
Time [μs]
g
D
Norm. PL intesnity
Frequency [GHz]
extent of linear slope
δω
h
Spin-coated NDs
Temperature change in air
i
Glass
ND-labeled worms
Agar pad
with buffer
Pipette
FCCP or DMSO
Temperature change in vivo
Biochemical stimuli

Fig. S1: **Experimental setup.** (a) Schematic of the experimental setup for the optical layout and microwave circuit. NDF: neutral density filter. LLF: laser line filter. HWP: half-wave plate. L: lens. DBS: dichroic beam splitter. LPF: long pass filter. CCD: charge-coupled device camera. APD: avalanche photodiode. SPA: spectrum analyzer. MW: microwave source. DAQ: data acquisition board. (b) Geometry of electromagnetic simulation of microwave linear antenna based on a finite element method. (c) Calculated magnetic field intensity map. (d) One-dimensional cross-sectional plot of the simulated magnetic field along the yellow-dashed line of Fig. S1c for the media of air and water. The input power is set to 11 dBm. The distance is from the antenna surface. The shaded area and vertical dashed line indicate the wire region and the measurement area (20 $\mu\mathrm{m}$) of the *in-vivo* measurements, respectively. The solid lines represent the exponential fit to the profile, giving the penetration depth of 40 $\mu\mathrm{m}$ for both of the media. (e) Pulse control sequences for CW-ODMR measurements and (f) four-point measurements. 532: green laser. MW: microwave. Sig: signal for $I_{\mathrm{PL}}^{\mathrm{ON}}$. Ref: reference for $I_{\mathrm{PL}}^{\mathrm{OFF}}$. ω_1 to ω_4 are the four frequencies used for the four-point measurements. (g) ODMR spectra fitted using the sum of two Lorentzians centered at the zero-field splitting frequency D and two linear functions to the slopes. Selected frequencies of ω_1 to ω_4 and their intermediates, ω_- and ω_+), are indicated by orange spheres and are separated on each slope by $\delta\omega$, as shown in the inset. (h) Schematic of NDs on coverslip in air and (i) of ND-labeled worms.

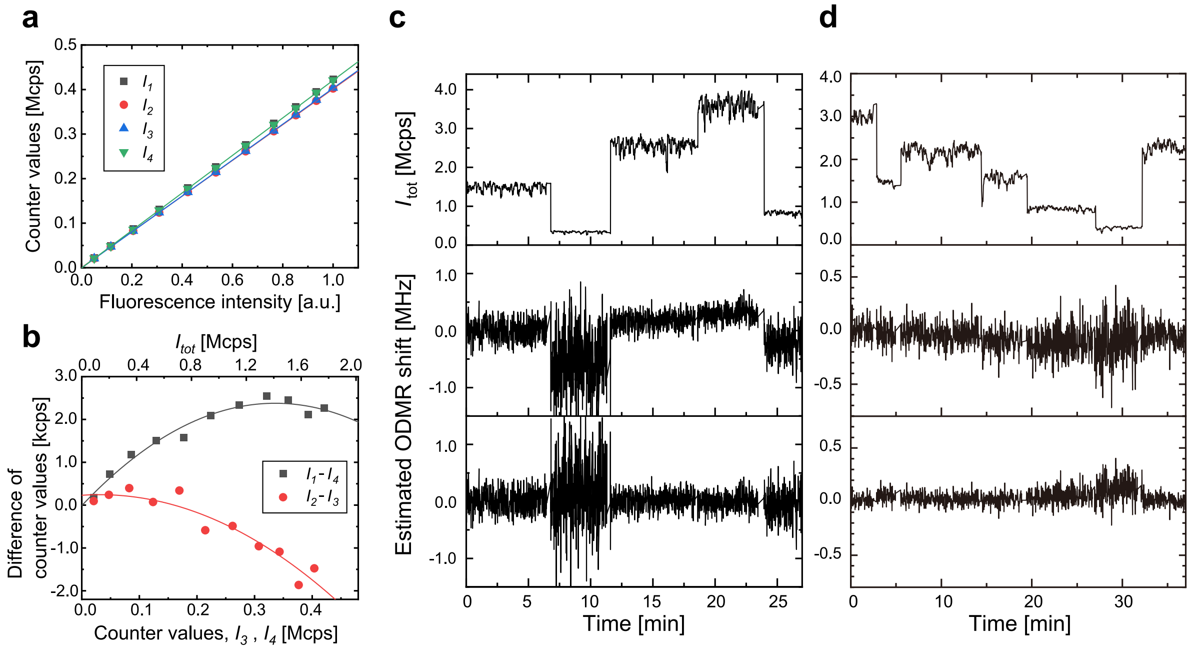

Fig. S2: **Variations of photo-responsivity of counters and the error-correction results.** (a) Photon counts of each counter from I_1 to I_4 as a function of ND fluorescence with their linear fits. I_1, I_4 and I_2, I_3 show similar increases reflecting the difference in the ODMR contrast. However, a very small difference was observed between I_1 and I_4 (I_2 and I_3) as an instrumental artifact. (b) Difference in the counter values between the two sets of counters, namely $I_1 - I_4$ and $I_2 - I_3$, as functions of I_4 and I_3, respectively. Solid lines represent second-order polynomial fits to the data. (c) Time profiles of photon counts of all the counters (I_{tot}, top) over 27 min with intentional variations in NV fluorescence intensity by the laser intensity control (top). The corresponding time profiles of the estimated ODMR shift without (middle) and with (bottom) the error correction. The sampling time is 500 ms. T_{S} is set at 36 °C. (d) Time profiles of photon counts of all the counters (I_{tot}, top) over 27 min with intentional variations in NV fluorescence intensity by varying the fluorescence intensity control (top). The corresponding time profiles of the estimated ODMR shift without (middle) and with (bottom) the error correction. The sampling time is 500 ms. T_{S} is set at 36 °C.

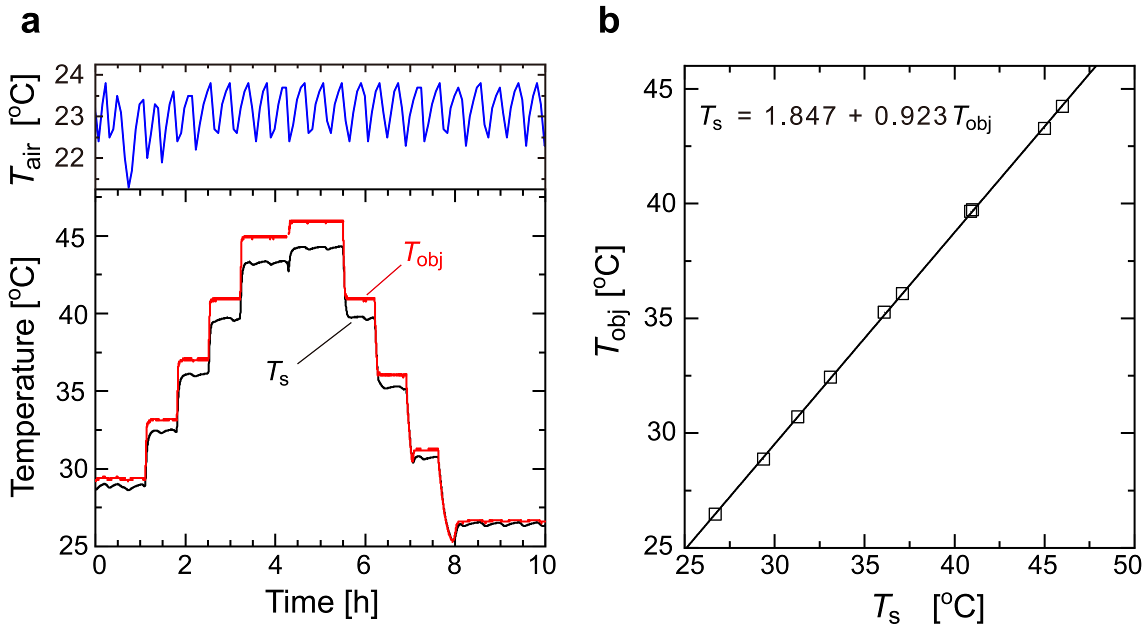

Fig. S3: **Calibration of the sample temperature (T_S) with that of the microscope objective (T_{obj}).** (a) Temperature profiles of room temperature (T_{air}, blue), T_{obj} (red), and T_S over 10 h. (b) The obtained relation between T_{obj} and T_S with the linear fit. $T_S = 1.847 + 0.923T_{obj}$ is obtained.

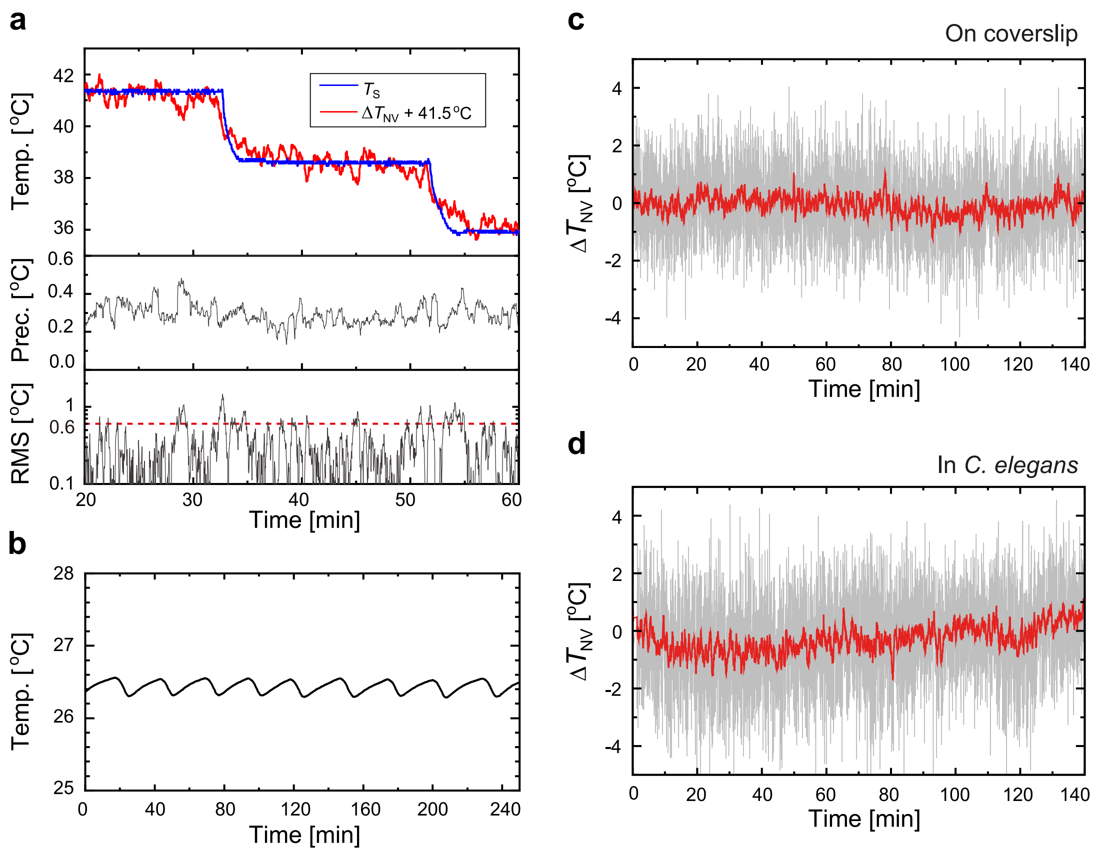

Fig. S4: **Accuracy and precision of the ND quantum thermometry and the temperature stability.** (a) Time profiles of temperatures for moving-averaged $\Delta T_{\rm NV}$ and $T_{\rm S}$ (top; Temp.), precision (middle; Prec.) and RMS of $T_{\rm S} - T_{\rm NV}$ (bottom; RMS) for the time-frame between 20 and 60 min of Fig. 2b. The red dashed line indicates the accuracy threshold. (b) Stability of the incubator temperature measured by the thermistor. The 30-min periodic oscillations are caused by the room temperature fluctuation. (c) Stability of $\Delta T_{\rm NV}$ for NDs on the coverslip at 36 °C and (d) in *C. elegans* at 23 °C. Gray: 1-s sampling data. Red: moving averaged data of 20 sampling points.

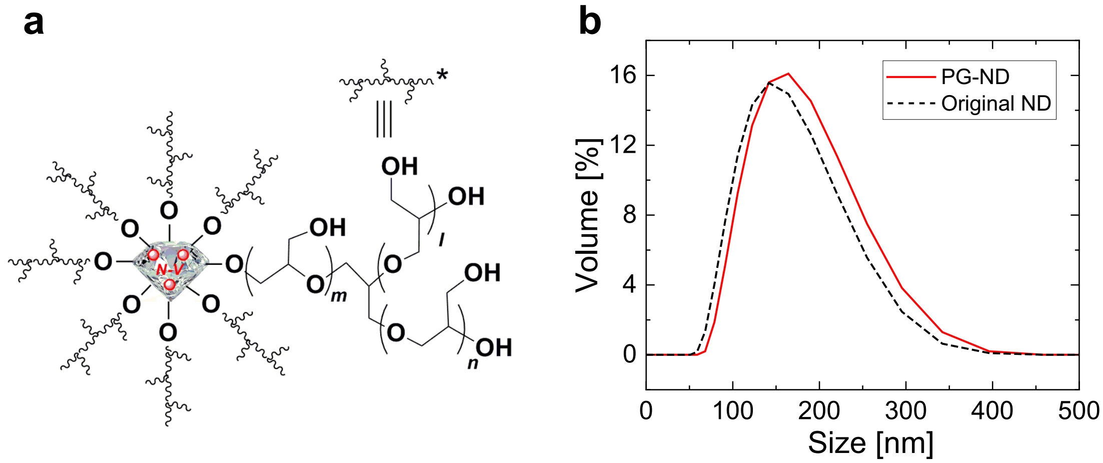

Fig. S5: **Properties of PG-NDs.** (a) Schematic of PG-NDs and (b) its dynamic light scattering data with that of original NDs before the surface functionalization.

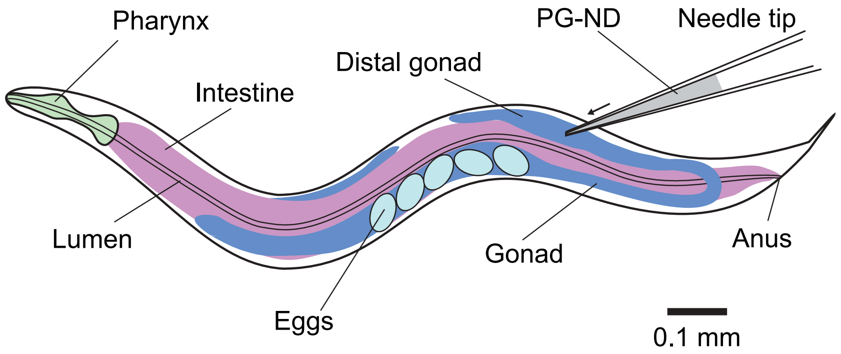

Fig. S6: **Anatomy of an adult *C. elegans* for microinjection of PG-NDs into the gonad.** Anatomical structure of an adult hermaphrodite, left lateral side. The scale bar indicates 0.1 mm. PG-ND suspension was microinjected into the distal arm of the gonad. PG-NDs were dispersed in the distal gonad and oocytes.

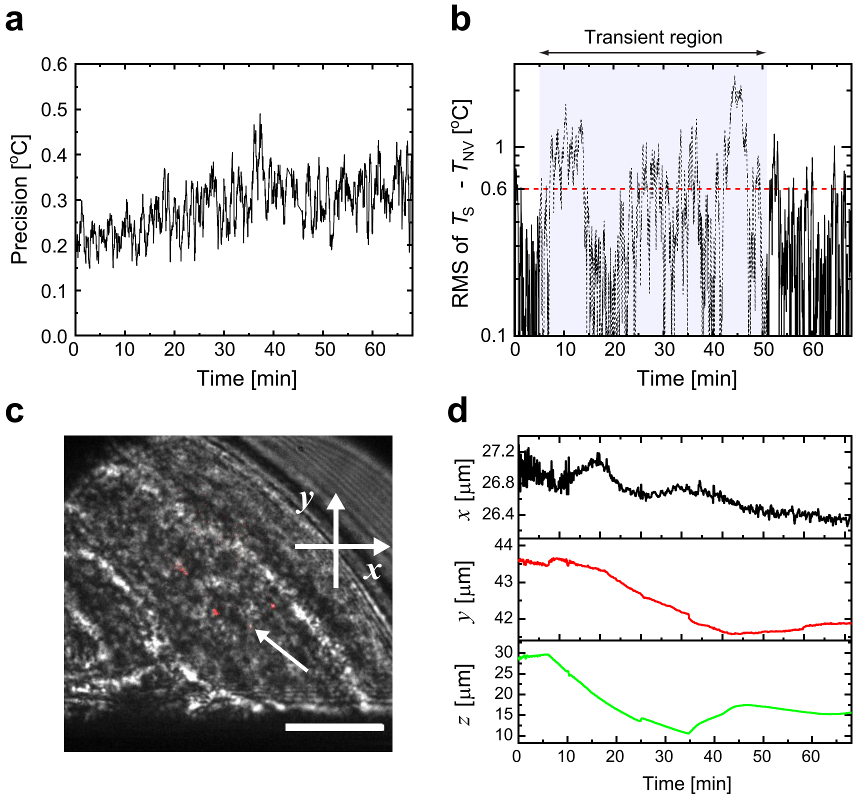

Fig. S7: **Precision, accuracy, and positional data of *in-vivo* thermometry.** Time profiles of (a) precision (20 sampling points) and (b) of the RMS of $T_\mathrm{S} - T_\mathrm{NV}$. (c, d) Photograph showing the xyz axes and time profile of the positional tracking in the xyz axes during the measurement. The z-axis is perpendicular to the paper. Scale bar: 20 µm.

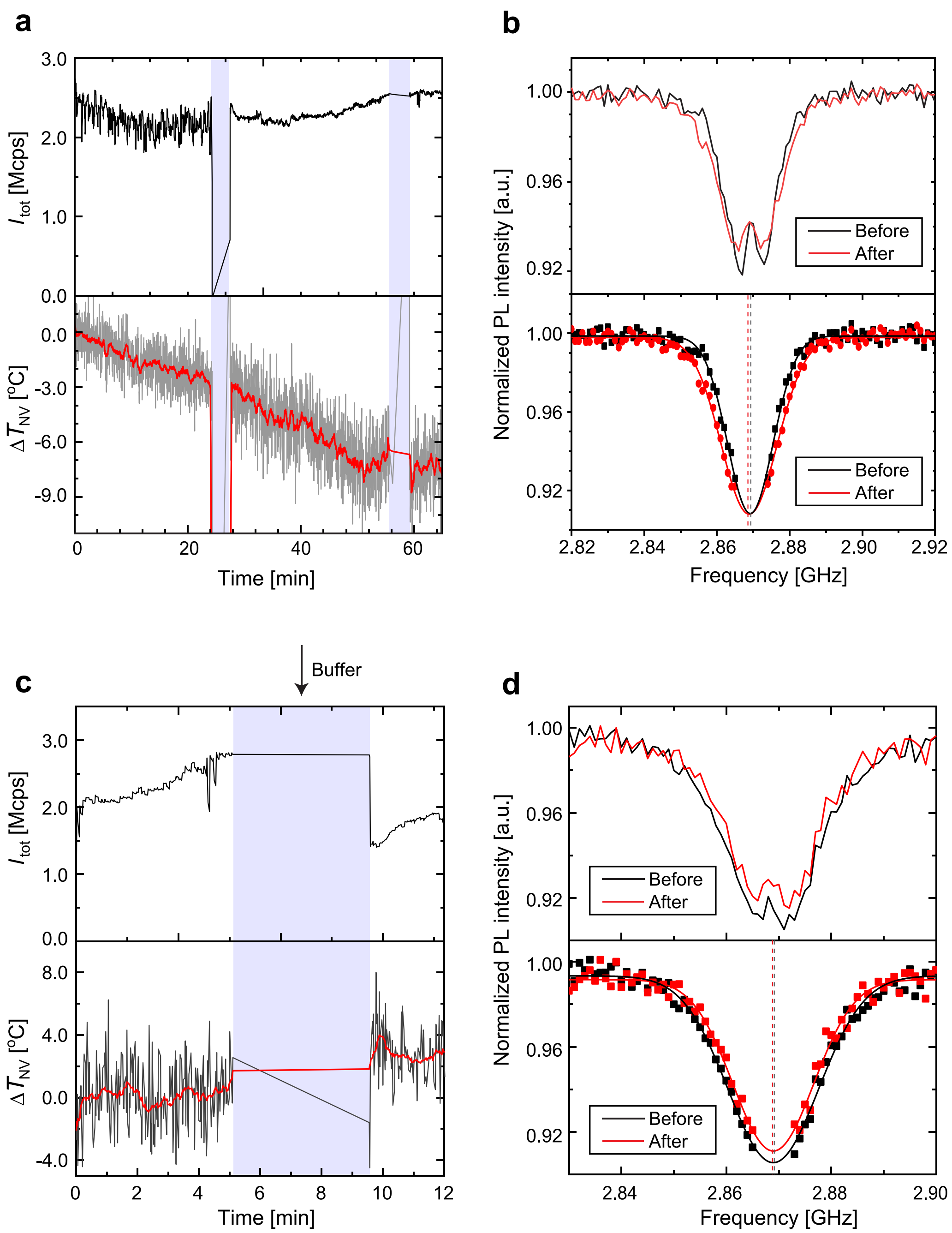

Fig. S8: **Detailed analysis of the baseline drifts and jumps of ΔT_{NV} inside worms.** (a) Time profile of ΔT_{NV} over 70 min. The blue shaded regions are periods during which the temperature measurements were not performed. (b) CW-ODMR spectra before and after the baseline drift with Gaussian fitting. The peak splitting structures are omitted to enable curve-fitting as described in Ref. 21. (c) Time profile of ΔT_{NV} when the baseline jump occurs after the addition of buffer. The baseline jumps only occur after the droplet addition. (d) CW-ODMR spectra before and after the baseline jumps with the Gaussian fitting.

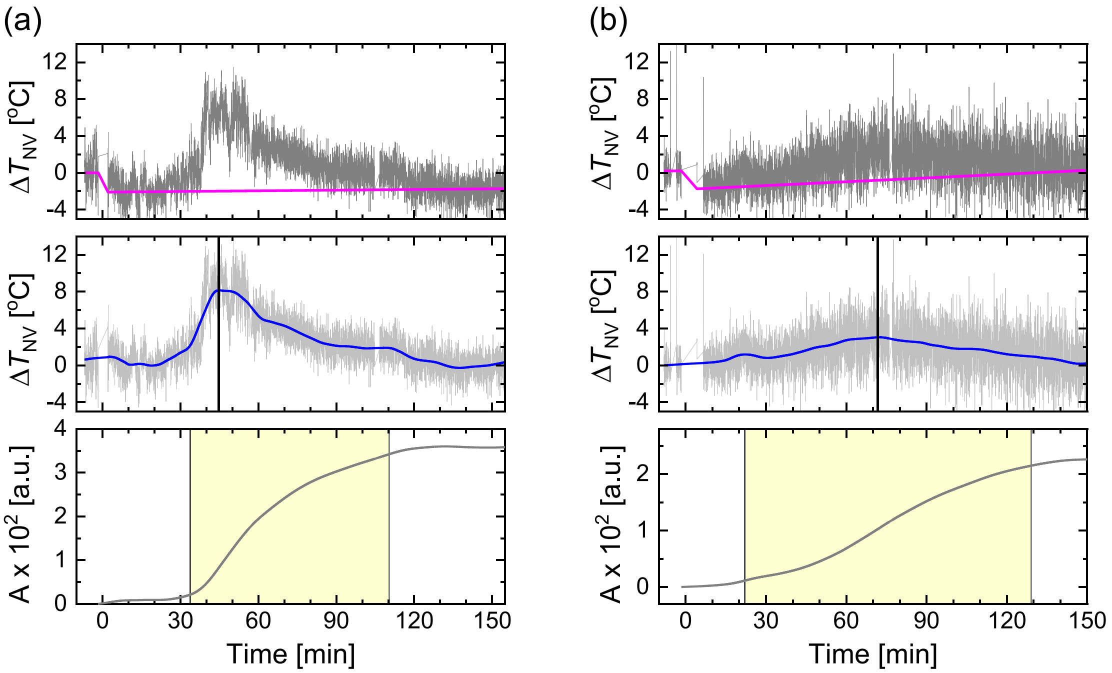

Fig. S9: **Graphical explanation of the quantifying method to determine the characteristic parameters of the response curves for two representative cases in the FCCP stimulation experiment (a, b).** In these figures, time zero indicates the time the FCCP drop was added. (Top) Original response curves (gray) with subtracted baseline drift (magenta). (Middle) The subtracted data (light gray) with the smoothing profile of the Lowess filter (navy). The vertical black lines indicate the maximum temperature points. (Bottom) Integrated area A showing 5 through 95 % integration area is shaded. Time points representing 5 % increase in integration are used to determine latency. The elapsed time between the 5 and 95 % integration time points is used as the duration.

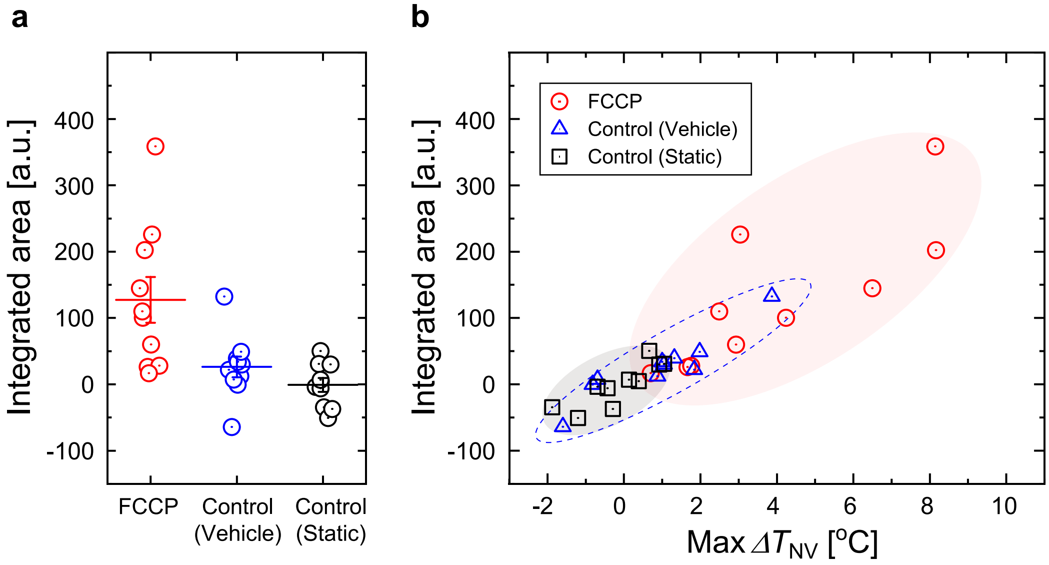

Fig. S10: **Additional statistical analysis of the temperature response curves.** (a) Statistical plots of the integrated areas of the response curves for FCCP stimulation, vehicle control, and static control (no solution added). n = 10 for all the data. The mean values of the integrated areas (with standard error) are (128 ± 35), (26 ± 16), and (-1 ± 10) for the FCCP, vehicle control, and static control experiments, respectively. (b) Correlation plots between the maximum ΔT_{NV} and the integrated areas of the temperature response curves for the three cases: FCCP addition (red circles), vehicle control (addition of vehicle without FCCP; blue triangles), and static control (no solution added; black boxes). Clearly, there are three clusters representing the above three types of experiments. The wide distribution of FCCP is prominent probably because of insufficient controllability of the FCCP concentration or dehydration-induced viability.
